# Parametric Optimization of FDM Process for PA12-CF Parts Using Integrated Response Surface Methodology, Grey Relational Analysis, and Grey Wolf Optimization

**DOI:** 10.3390/polym16111508

**Published:** 2024-05-27

**Authors:** Ali Saeed Almuflih, Muhammad Abas, Imran Khan, Sahar Noor

**Affiliations:** 1 Department of Industrial Engineering, College of Engineering, King Khalid University, Abha 61421, Saudi Arabia; 2 Center for Engineering and Technology Innovations, King Khalid University, Abha 61421, Saudi Arabia; 3 Department of Industrial Engineering, University of Engineering and Technology Peshawar, Peshawar 25120, Pakistan; sahar@uetpeshawar.edu.pk; 4 Department of Mechanical Engineering, University of Engineering and Technology Peshawar, Peshawar 25120, Pakistan; engrimran@uetpeshawar.edu.pk

**Keywords:** additive manufacturing, fused deposition modeling, carbon fiber-reinforced polyamide 12, response surface methodology, grey relational analysis, grey wolf optimization

## Abstract

Efficiently managing multiple process parameters is critical for achieving optimal performance in additive manufacturing. This study investigates the relationship between eight key parameters in fused deposition modeling (FDM) and their impact on responses like average surface roughness (Ra), tensile strength (TS), and flexural strength (FS) of carbon fiber-reinforced polyamide 12 (PA 12-CF) material. The study integrates response surface methodology (RSM), grey relational analysis (GRA), and grey wolf optimization (GWO) to achieve this goal. A total of 51 experiments were planned using a definitive screening design (DSD) based on response RSM. The printing process parameters, including layer thickness, infill density, and build orientation, significantly affect Ra, TS, and FS. GRA combines responses into a single measure, grey relational grade (GRG), and a regression model is developed. GWO is then employed to optimize GRG across parameters. Comparison with GRA-optimized parameters demonstrates GWO’s ability to discover refined solutions, reducing average surface roughness to 4.63 μm and increasing tensile strength and flexural strength to 88.5 MPa and 103.12 MPa, respectively. Practical implications highlight the significance of GWO in industrial settings, where optimized parameters lead to reduced costs and improved product quality. This integrated approach offers a systematic methodology for optimizing FDM processes, ensuring robustness and efficiency in additive manufacturing applications.

## 1. Introduction

Additive Manufacturing (AM) techniques, particularly fused deposition modeling (FDM), have gained remarkable attention in recent years owing to their versatility, cost-effectiveness, rapid prototyping, and functional parts capabilities with good properties [1]. Their applications have been explored in aerospace, automobile, medical, biomedical, and electronic fields [2,3,4,5,6,7,8].

However, achieving optimal performance and quality in FDM processes necessitates tuning various parameters. For this aim, a systematic approach based on the design of experiments (DOE) such as Taguchi’s orthogonal array design, response surface methodology (RSM) integrated with artificial neural networks (ANN), evolutionary algorithms, and multi-criteria decision-making methods (MCDM) have been successfully applied. For instance, Muhamedagic et al. [9] analyzed key process parameters affecting tensile strength in FDM-printed parts using a short carbon fiber-reinforced polyamide composite. Employing RSM and ANN, the study identified layer thickness and raster angle as the most influential factors. RSM established a reduced cubic model, while ANN determined the optimal configuration for predicting tensile strength, offering time efficiency through k-fold cross-validation. Fountas et al. [10] investigated the flexural strength of PET-G by varying FDM process parameters. They conducted an RSM with 27 runs. Analysis of variance (ANOVA) generated a full quadratic regression equation, later implemented as an objective function of the grey wolf algorithm (GWO). The GWO algorithm suggested a parameter combination that improved flexural strength by approximately 15% compared to the highest value obtained from experimental runs. The study by Nagendra et al. [11] focused on enhancing FDM by introducing nylon with added aramid short fibers for functional parts. The Gray Taguchi technique optimizes FDM parameters. Results demonstrate improved properties over pure nylon: 7.2% higher tensile strength, 22.7% higher flexural strength, 27.4% higher impact strength, and 7.5% higher compressive strength, validating the feasibility of short fiber composites in FDM. Balaji et al. [12] examined the ePA-CF filament’s mechanical properties, varying layer thickness (LT), raster angle (RA), and infill density (ID). ANN analysis identified the key parameters. Printing with LT = 0.14 mm, ID = 100%, and RA = 90∘ resulted in enhanced tensile strength (66 MPa), flexural strength (87 MPa), and impact strength (12.5 KJ/m^2^). Kumar et al. [13] optimized surface roughness (SR), production time (PT), and volume percentage error (VPE) in FDM. They employed a parametric optimization technique integrating ANN and Whale Optimization Algorithm (WOA). An ANN model was developed using experimental data, serving as an objective function in WOA to minimize output responses. The method’s robustness was successfully validated on optimal FDM process parameter combinations. Saad et al. [14] employed RSM, particle swarm optimization (PSO), and symbiotic organism search (SOS) to enhance the surface quality of FDM printed parts. RSM guided the experimental design, establishing a regression model linking input parameters to surface roughness. Validated model accuracy enabled coupling with PSO and SOS for optimizing parameters and minimizing surface roughness. PSO and SOS improved surface roughness by approximately 8.5% and 8.8%, respectively, compared to the conventional RSM method. Chinchanikar et al. [15] employed various optimization techniques, including technique for order of preference by similarity to ideal solution (TOPSIS), desirability function-based RSM, non-dominated sorting genetic algorithm (NSGA-II), and GRA to determine optimal FDM process parameters for tensile, impact, flexural, and surface roughness. The study highlights the superior prediction accuracy achieved through a hybrid optimization approach, specifically the combination of a genetic algorithm (GA) with RSM. Salunkhe et al. [16] studied the relationship between PLA material’s tensile strength and 3D printing parameters (infill density, layer height, print speed, and extrusion temperature) in FDM. Six optimization methods were used: cohort intelligence (CI), PSO, GA, teaching–learning-based optimization (TLBO), simulated annealing (SA), and JAYA, which yielded the highest tensile strength. Boppana and Ali [17] improved the tensile strength of polycarbonate (PC) samples printed using FDM by employing an integrated approach of I-optimal design, ANN, and GA techniques. Mohanty et al. [18] optimized the dimensional accuracy of FDM-printed parts using 10 different metaheuristic approaches, namely GA, SA, PSO, GWO, moth flame optimization (MFO), WOA, JAYA, sunflower optimization algorithm, Lichtenberg algorithm optimization, and forensic-based investigation optimization. The results showed that all approaches performed similarly and provided optimal settings. Various other studies [19,20,21,22,23,24,25] have explored the optimization of process parameters in additive manufacturing using hybrid approaches. These studies utilized various intelligent algorithms to attain optimal results for specific objectives related to their respective problems.

As discussed above, various metaheuristic approaches are employed for the optimization of process parameters for FDM. Each of these methods offers unique strategies for navigating the complex parameter space of FDM processes to achieve optimal performance and quality. GWO distinguishes itself from other optimization approaches in terms of parameter simplicity. Compared to PSO, GA, SA, NSGA II, WAO, TLBO, and JAYA, GWO usually requires fewer parameters to be tuned. While algorithms like PSO and GA often involve tuning parameters such as inertia weight and crossover probability, GWO’s hierarchical structure and hunting mechanism streamline the optimization process, reducing the need for extensive parameter optimization. This simplicity not only makes GWO easier to implement but also lessens the burden of parameter tuning, making it more accessible to practitioners. By minimizing the number of parameters, GWO offers a more straightforward and efficient optimization solution, particularly suitable for scenarios where simplicity and ease of use are paramount [26]. The GWO algorithm also provides a good balance between exploration and exploitation, especially in search spaces with a large number of local optima. As a result of its continual reduction of search space and a limited number of variables to decide, the GWO does not need large storage and has fast convergence [27].

The review of existing literature highlights that effectively managing multi-objective process parameters is crucial for achieving optimal performance, yet it remains challenging. This study explores the relationship between eight key FDM process parameters and their impact on response variables such as average surface roughness (Ra), tensile strength, and flexural strength of carbon fiber-reinforced polyamide 12 (PA 12-CF) material. Parameters include layer thickness, number of perimeters, infill density, printing speed, fill angle, bed temperature, extrusion temperature, and build orientation. A definitive screening design (DSD) based on response surface methodology is employed, comprising 51 experiments to gather necessary data for further analysis. Grey relational analysis (GRA) is utilized to consolidate multi-responses into a single measure using grey relation grade (GRG). A regression model is then developed, exhibiting a strong correlation and predictability of GRG. Subsequently, this regression model serves as the objective function for optimizing GRG across the considered parameters using the grey wolf optimization (GWO) algorithm. Despite limited exploration of GWO in FDM parametric optimization, this study proposes it as a novel approach for enhancing FDM-processed PA 12-CF specimens.

## 2. Materials and Methods

A commercial-grade filament known as polyamide 12 reinforced with carbon fiber (PA 12-CF) supplied by eSUN (Shenzhen, China) is used in the present study as it is utilized for various industrial applications. The manufacturer’s specifications indicate that 15% carbon fiber is added to enhance the strength, rigidity, and toughness of nylon, providing an effective substitute for metal in many scenarios. This filament exhibits a low water absorption rate and is less affected by changes in humidity and temperature, ensuring consistent dimensions when printing parts. Its self-lubricating and wear-resistant properties make it particularly suitable for printing gears, offering durability and reliability in mechanical applications. Moreover, PA 12-CF demonstrates high-temperature resistance, with parts capable of continuous use at temperatures of up to 120 °C, and short-term use at temperatures reaching 160 °C. Its low shrinkage during printing minimizes the risk of warping and cracking, while the resulting printing surface is matte and delicate.

The filament has a diameter of 1.75 mm and a density of 1.24 g/cm^3^. Before printing, the filament is dried at 70 °C for 12 h to achieve the best printing. To enhance bed adhesion, a thin layer of polyvinylpyrrolidone (PVP) solid glue is applied to the print bed before starting to print. This layer acts as a temporary adhesive, helping to keep the initial layers of filament firmly in place during printing. As the print progresses, the filament adheres to the glue layer, providing a stable foundation for the rest of the print. Further, it is non-toxic and fumeless.

**Figure 1:**
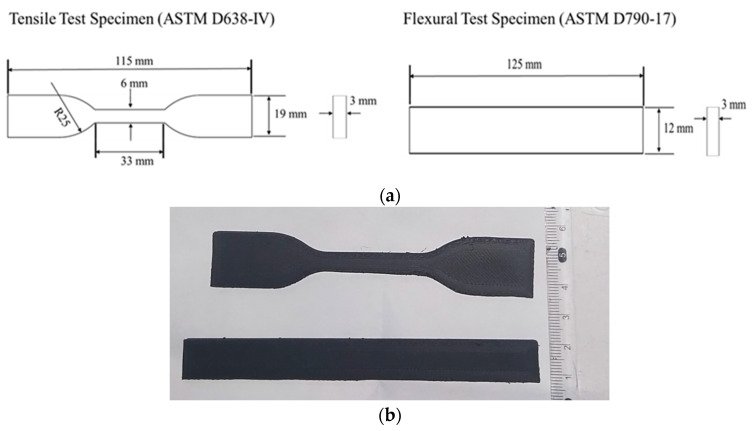
(**a**) Schematic of tensile and flexural test specimens according to ASTM standards and (**b**) printed specimens.

The test specimens were printed according to ASTM standards i.e., ASTM D638-IV [28] for tensile test and ASTM D790-17 [29] for the flexural test, as shown in Figure 1.

### 2.1. Printing Process Parameters and Experimental Design

The printing process parameters considered in the present study are layer thickness (LT), number of contours/perimeters (NC), infill density (ID), fill angle (FA), printing speed (PS), nozzle/extrusion temperature (ET), bed temperature (BT), and build orientation (BO). Each of these parameters plays an important role in determining the quality, strength, and accuracy of the printed object. Layer thickness influences the resolution and surface finish of the print, while the number of contours or perimeters affects its structural integrity. Infill density determines the internal strength and weight of the object, while fill angle optimizes strength in specific directions. Printing speed directly impacts production time and can affect print quality, especially on complex designs. Nozzle or extrusion temperature and bed temperature are critical for ensuring proper material flow and adhesion between layers. Built orientation is a strategic consideration, influencing factors such as support material usage, print time, and structural strength.

The three-level definitive screening design (DSD) based on response surface methodology (RSM), developed by Jones and Nachtsheim [30,31], is utilized for experimental runs. This design has the ability to seamlessly integrate screening and response surface optimization into a single framework, a feature not commonly found in traditional screening designs. One of the key advantages of the DSD is its ability to evaluate the main effects, interaction effects, and quadratic effects efficiently, even with a limited number of experimental runs. This streamlined approach significantly reduces the resources and time required for experimentation, while providing comprehensive insights into the underlying process dynamics. The efficacy of the DSD in optimizing and constructing predictive models for the FDM process has been extensively demonstrated in previous studies [32,33,34,35,36,37]. These studies collectively validate the reliability and versatility of the DSD in identifying critical process parameters and their interactions, thus facilitating the development of optimized printing conditions and improved product quality.

The levels for eight printing process parameters are tabulated in Table 1, and the experimental plan based on DSD is tabulated in Table 2. The DSD includes a total of 17 experimental runs; however, each experiment is randomly repeated three times to account for the variability inherent in the printing process. This results in a total of 51 experimental runs.

### 2.2. Responses

The responses considered in the present study are average surface roughness (Ra), tensile strength (TS), and flexural strength (FS). Their values, which are obtained based on DSD experimental runs, are tabulated in Table 3. Tensile and flexural tests were performed on the universal testing machine (5567, INSTRON, Wenling, Zhejiang, China). The tensile test was performed at a crosshead speed of 5 mm/min and the flexural test was performed at 1.3 mm/min at room temperature (25 ± 5 °C).

The Mitutoyo surface roughness tester (SJ-201, Mitutoyo Corporation, Takatsu-ku, Kawasaki, Kanagawa, Japan) was used to measure Ra using the stylus method, adhering to the guidelines outlined in the ISO 21920 industrial standards [38]. To ensure reliability and representativeness, five readings were taken perpendicular to the printed layers of each specimen. This approach mitigates variations in surface roughness within the printed layers. The specific settings of the Mitutoyo surface roughness tester included an evaluation length of 4 mm, a tip angle of 90°, a tip radius of 2 µm, a tracing speed of 0.25 mm/s, and a cut-off wavelength of 0.8 mm. These standardized settings were employed to maintain consistency and accuracy throughout all measurements.

### 2.3. Multi-Response Optimization

For multi-response optimization, an integrated approach of grey relational analysis (GRA) and the metaheuristic technique, namely grey wolf optimization (GWO), is employed. Initially, the multi-responses are converted to a single response using GRA. Subsequently, the regression model is created and further optimized using GWO.

### 2.4. Grey Relational Analysis

Grey relational analysis (GRA) is a method used for analyzing the relationships between multiple factors or responses within a dataset. It was developed by Deng in the late 1980s [39] and has since been applied in various fields such as engineering, economics, and decision-making [40,41,42]. The fundamental principle of GRA lies in its ability to assess the similarity or correlation between different responses, even when they are measured using different units or scales. GRA accomplishes this by transforming the original data into dimensionless grey relational grades, which represent the degree of resemblance or proximity between each pair of responses. GRA is particularly useful in situations where traditional statistical methods may not be applicable due to the presence of non-linear relationships. By capturing the inherent relationships between responses, GRA enables analysts to make informed decisions and optimize processes in diverse fields ranging from engineering design to financial analysis. Its flexibility and robustness make it a valuable tool for tackling complex optimization problems in real-world scenarios [43].

The process of GRA typically involves the following steps:

Normalization: In this step, the responses obtained for each experimental run are normalized to a comparable scale. This ensures that responses with different units or scales are brought to a common level, allowing for meaningful comparison. For minimization of response, Equation (1) is used, and for maximization of response, Equation (2) is used.

(1) $${µ}_{i}=\frac{max\;y_{i}-y_{i}}{max\;y_{i}-min\;y_{i}}$$

(2) $${µ}_{i}=\frac{y_{i}-{min\;y}_{i}}{max\;y_{i}-min\;y_{i}}$$

where $\mu_{i}$ is the normalized value for the $i^{th}$ experiment, $y_{i}$ is the individual value of measured response at experiment number *i*, and $max\;y_{i}$ and $min\;y_{i}$ are the maximum and minimum values of experimental data obtained for the response.

Calculation of Grey Relational Coefficients: Once the data is normalized, grey relational coefficients are computed to quantify the relationship between each pair of responses. This is achieved by comparing the normalized values of each response across all observations and determining the degree of similarity or resemblance using Equation (3).

(3) $$\delta_{i}=\frac{\eta_{min}+€\eta_{max}}{\eta_{0,i}\left(j\right)-€\eta_{max}}$$

where $\delta_{i}$ is the GRC value at experiment i, $\eta_{0,i}$ is the deviation from the target value and can be calculated using Equation (4), $\eta_{min}$ and $\eta_{max}$ are the minimum and maximum values $\eta_{0,i}\left(j\right)$, and € is the distinguish/identification coefficient value, which is fixed at 0.5 for the present study.

(4) $$\eta_{0,i}\left(j\right)=1-\mu_{i}(j)$$

Determination of Grey Relational Grades: The grey relational coefficients obtained in the previous step are used to calculate the grey relational grades for each response using Equation (5). These grades represent the overall similarity or proximity of each response to the others within the dataset.

(5) $${£}_{i}=\frac{1}{n}\sum_{j=1}^{n}{w_{j}\delta }_{i}$$

where ${£}_{i}$ is weighted GRG for the $i^{th}$ experiment and $w_{j}$ is the normalized weightage of response j.

Ranking and Analysis: Finally, the average values of GRG are ranked in descending order, with GRG having the highest average values corresponding to the best experimental run. This ranking provides the relative importance of each response and helps in decision-making or optimization processes.

### 2.5. Grey Wolf Optimization (GWO)

Grey wolf optimization (GWO) is a metaheuristic optimization algorithm that was first proposed by Mirjalili et al. in 2014 [44]. The algorithm is inspired by the social hierarchy and hunting behavior of grey wolves in the wild. GWO is used to find the global minimum or maximum of a function with a large number of unknown parameters, called the optimization problem.

In GWO, the search space is divided into three positions, which correspond to the alpha, beta, and delta wolves. These positions represent the most promising solutions found by the swarm at any given time. Initially, the positions of the alpha, beta, and delta wolves are randomly initialized in the search space.

**Figure 2:**
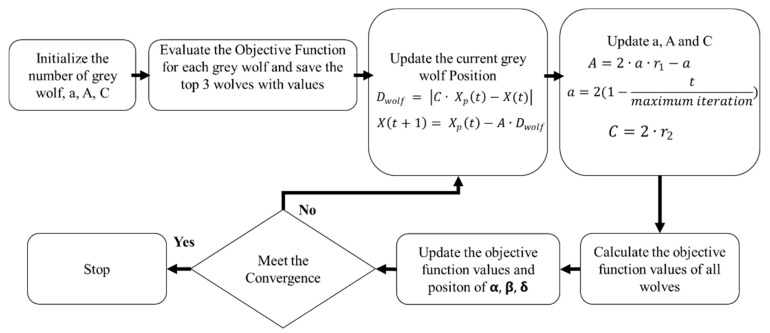
Flow chart for GWO.

Figure 2 illustrates the flow chart for GWO. At the start of the algorithm, the fitness of each wolf’s position is evaluated using the objective function that needs to be optimized. The objective function assigns a fitness value to each wolf, which represents how close it is to the optimal solution. The wolf with the best fitness value is called the alpha wolf (α), the wolf with the second-best fitness value is called the beta wolf (β), and the wolf with the third-best fitness value is called the delta wolf (δ). The distance of each wolf from the positions of the alpha, beta, and delta wolves is computed using Equation (6). The position of each wolf is updated based on its distance from the alpha, beta, and delta wolves using Equation (7).

(6) $$D_{wolf}=\left|C\cdot X_{wolf}\left(t\right)-X(t)\right|$$

(7) $$X\left(t+1\right)=X_{wolf}\left(t\right)-A\cdot D_{wolf}$$

where t is the number of iterations, $X_{wolf}\left(t\right)$ represents the position of the grey wolf (α, β, δ), X(t) is the current position of the grey wolf, $X\left(t+1\right)$ is the next updated position of the grey wolf, and C and A are the coefficient vectors and can be determined using Equations (8) and (9).

(8) $$A=2\cdot a\cdot r_{1}-a$$

(9) $$C=2\cdot r_{2}$$

where $r_{1}$ and $r_{2}$ are random numbers between 0 and 1, a lies in the range 0–2 and decreases with the progression of the algorithm and can be computed using Equation (10)

(10) $$a=2(1-\frac{t}{maximum\;iteration})$$

The algorithm then updates the position of each wolf based on its current position and the positions of the alpha, beta, and delta wolves. The updated position of each wolf is a combination of its current position, a random term, and the positions of the alpha, beta, and delta wolves. The position update is based on the hunting behavior of the wolves, where the alpha wolf leads the pack, the beta wolf follows the alpha, and the delta wolf provides alternative solutions. The updated Equations (11)–(17) are as follows:

(11) $$D_{\alpha }=\left|C\cdot X_{\alpha }\left(t\right)-X(t)\right|$$

(12) $$D_{\beta }=\left|C\cdot X_{\beta }\left(t\right)-X(t)\right|$$

(13) $$D_{\delta }=\left|C\cdot X_{\delta }\left(t\right)-X(t)\right|$$

(14) $$X_{1}=X_{\alpha }\left(t\right)-A_{1}\cdot D_{\alpha }$$

(15) $$X_{2}=X_{\beta }\left(t\right)-A_{2}\cdot D_{\beta }$$

(16) $$X_{3}=X_{\delta }\left(t\right)-A_{3}\cdot D_{\delta }$$

(17) $$X\left(t+1\right)=\frac{X_{1}+X_{2}+X_{3}}{3}$$

The GWO algorithm continues to update the position of each wolf in the swarm until a stopping criterion is met. This criterion can be a fixed number of iterations or a certain level of convergence. In the present study, the number of iterations is used as the stopping criterion.

GWO’s performance depends on two key hyperparameters: the number of iterations and the number of wolves. For this aim, lists of values are defined at five levels for each parameter, and their different combinations are tested (by applying a nested loop in coding) to analyze the performance of the algorithm. The best combination that gives consistent and the highest objective function value is selected. The list of parameters and the levels are tabulated in Table 4. The five levels for the number of iterations likely aim to cover a range of durations for the optimization process. Starting from 100 iterations, which may represent a relatively quick optimization run, the levels increase in increments of 50, reaching up to 300 iterations. This choice allows for an exploration of how the GWO’s performance evolves with varying lengths of optimization cycles. Shorter iterations might offer quicker results but risk premature convergence, while longer iterations could potentially yield more accurate solutions at the expense of increased computational time. The five levels for the number of wolves, representing the population size in the optimization process, are chosen to span a range of population densities. Beginning with 10 wolves, which constitutes a smaller population, the levels increase by increments of 10, reaching up to 50 wolves. This selection enables an investigation into how the diversity and size of the population influence the GWO’s ability to explore the solution space effectively. A smaller population might lead to faster convergence but could also limit the algorithm’s ability to discover diverse solutions, while a larger population could potentially enhance exploration at the cost of increased computational resources.

## 3. Results and Discussions

### 3.1. Regression Analysis of Variance (ANOVA)

Regression analysis of variance (ANOVA) was conducted at a 95% confidence interval based on the definitive screening design (DSD) to evaluate the impact of process parameters on responses. Significance was determined by comparing *p*-values to an alpha value of 0.05, with values below indicating a significant effect. For average surface roughness (Ra) (Table 5), the results revealed that layer thickness (LT) had the most significant effect, contributing 52.36%, followed by build orientation (BO) at 28.23%. In contrast, fill angle (FA) exhibited minimal significance, with a contribution of only 0.07%. Moreover, the lack of fit was nonsignificant (0.634), indicating an adequate relationship between model terms and Ra.

For tensile strength (Table 6), the analysis highlighted the significant role of BO, with a contribution of 27.01%, followed by infill density (ID) and FA at 19.85% and 18.77%, respectively. Similarly, the lack of fit was negligible (0.696), affirming the adequacy of the model in capturing the relationship between model terms and tensile strength. This trend is reflected in the analysis of flexural strength (Table 7), where BO emerged as the most influential parameter, contributing 30.96%, followed by ID and FA at 19.29% and 16.96%, respectively. Once again, the lack of fit was nonsignificant (0.761), emphasizing the reliability of the model in explaining the variation in flexural strength.

**Figure 3:**
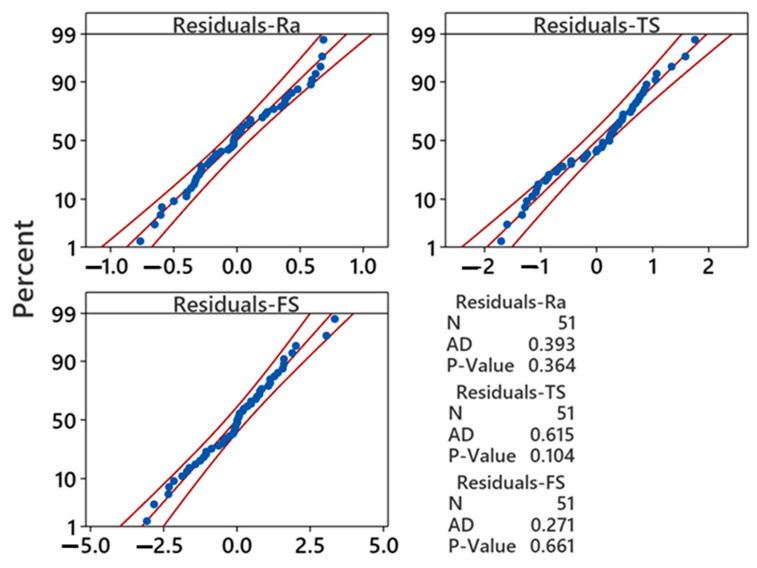
Normality plots for average surface roughness (Ra), tensile strength (TS), and flexural strength (FS).

The adequacy of the ANOVA results is analyzed using a normality plot of residuals and validated using the Anderson–Darling (AD) test. Figure 3 shows the normality plots, revealing that data points for all measured responses closely align with the middle fitted line. This observation suggests that the data follow normal distributions. To validate this assumption, the AD test—a robust statistical tool commonly utilized for outlier detection within normal distributions—was applied. The decision to accept or reject the null hypothesis regarding the normal distribution of the data depends on the *p*-value obtained from the AD test. Figure 3 shows that the *p*-values for each response exceeded 0.05. This indicates strong evidence supporting the null hypothesis and affirms that the collected data are indeed normally distributed. Accordingly, the data can be confidently utilized for further experimental analysis and optimization processes.

### 3.2. Effects of Process Parameters on Responses

**Figure 4:**
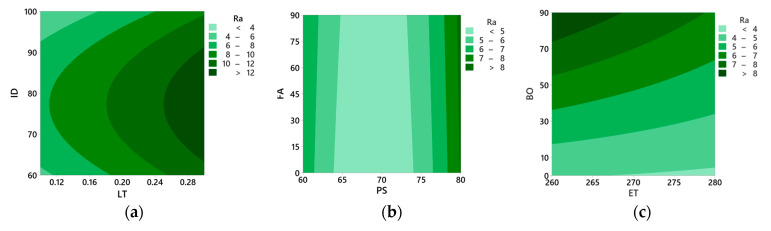
Contour plots for average surface roughness (Ra). (**a**) infill density vs. layer thickness (**b**) fill angle vs. printing speed, and (**c**) build orientation vs. extrusion temperature.

**Figure 5:**
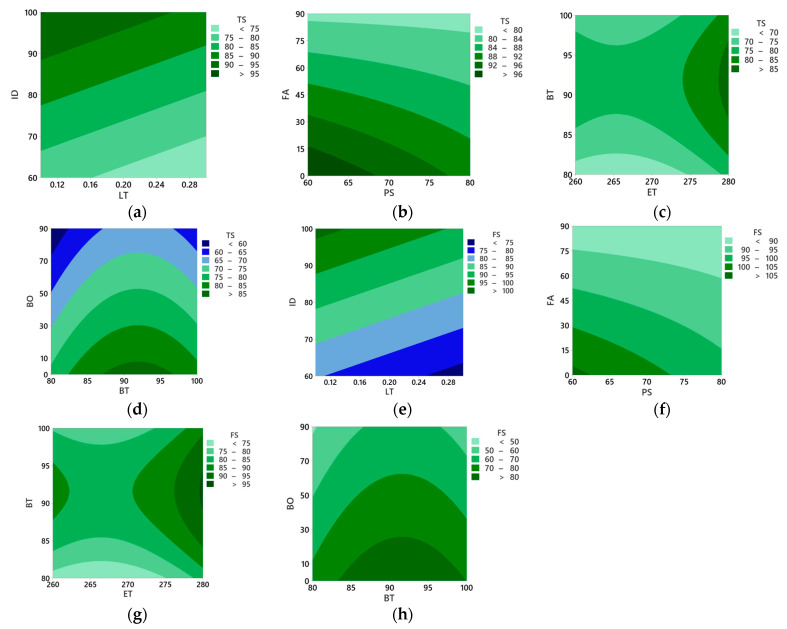
Contour plot for (**a**) tensile strength, infill density vs. layer thickness, (**b**) tensile strength, fill angle vs. printing speed, (**c**) tensile strength, bed temperature vs. extrusion temperature, (**d**) tensile strength, build orientation vs. bed temperature, (**e**) flexural strength, infill density vs. layer thickness, (**f**) flexural strength, fill angle vs. printing speed, (**g**) flexural strength, bed temperature vs. extrusion temperature, and (**h**) flexural strength, build orientation vs. bed temperature.

The effects of process parameters on average surface roughness (Ra), tensile strength (TS), and flexural strength (FS) were studied using contour plots, as illustrated in Figure 4 and Figure 5.

#### 3.2.1. Effects of Process Parameters on Ra

Figure 4a shows that the lower layer thickness and higher infill density have lower Ra. Larger layer height results in a rougher surface finish because each layer is thicker, leading to a more noticeable stair-stepping effect [45]. With high infill density, a dense structure is formed with fine layers adhering to each other, thus reducing porosity and providing a smooth surface [46]. Figure 4b shows that the printing speed between 65 mm/s and 75 mm/s results in lower Ra; however, the fill angle has no pronounced effect. Higher printing speeds can lead to a rougher surface finish due to less time for each layer to cool and solidify properly, potentially causing more imperfections [47]. Figure 4c illustrates that higher extrusion temperatures and build orientation of 0° result in lower Ra. Higher extrusion temperatures ensure better adhesion between layers and minimize imperfections between layers, thereby improving the surface quality [48]. An increase in build orientation causes overhangs or steep angles that may result in poorer surface quality and a prominent staircase effect [49].

#### 3.2.2. Effects of Process Parameters on Tensile and Flexural Strengths

Figure 5a,e shows that at lower layer thickness and higher infill density, the tensile and flexural strength values are higher. This is because larger layer thickness typically results in weaker parts due to reduced layer adhesion that causes voids [50], while higher infill densities provide more material within the structure to bear loads [51]. Figure 5b,f illustrates that lower printing speed and infill angle result in higher tensile and flexural strength. Higher printing speeds often result in weaker parts as layers may not have sufficient time to bond adequately [52]; however, a larger fill angle may weaken the structure’s integrity by altering force distribution [53]. Figure 5c,g shows that higher extrusion temperatures and bed temperatures of approximately 90–95 °C have higher tensile and flexural strengths. Higher extrusion and bed temperatures improve layer adhesion and reduce warping, ultimately resulting in stronger parts [54,55]. Figure 5d,h shows that lower build orientations have higher tensile and flexural strength values. Increasing build orientation requires a support structure and is therefore more prone to printing defects, such as layer misalignment and delamination. These defects can introduce weak points and discontinuities in the printed part, compromising its tensile and flexural strengths [56,57].

### 3.3. Optimization Using Grey Relational Analysis (GRA)

The procedure for GRA is discussed in Section 2.4. As lower values of Ra are desirable, it is normalized using Equation (1). On the contrary, higher values are desirable for TS and FS, so they are normalized using Equation (2). Subsequently, grey relational coefficients (GRC) are computed according to Equations (3) and (4), as presented in Table 8. These coefficients are then transformed into grey relational grades (GRG) using Equation (5), outlined in the same table. Following this computational process, average GRG values are calculated for each level of every process parameter, as tabulated in Table 9. The highest average GRG value identified among the different levels signifies the optimal setting for the respective parameter. For instance, for layer thickness, the highest average GRG value, found at level −1, stands at 0.196, corresponding to a layer thickness of 0.1 mm. Analogously, optimal values are determined for other process parameters, including the number of perimeters (6), infill density (100%), fill angle (0°), print speed (70mm/s), extrusion temperature (280 °C), bed temperature (90 °C), and print orientation (0°). Consequently, optimal values for Ra, TS, and FS are obtained, measuring 5.55 µm, 85.45 MPa, and 98.36 MPa, respectively.

### 3.4. Regression Model Based on Grey Relational Grade (GRG) Values

**Figure 6:**
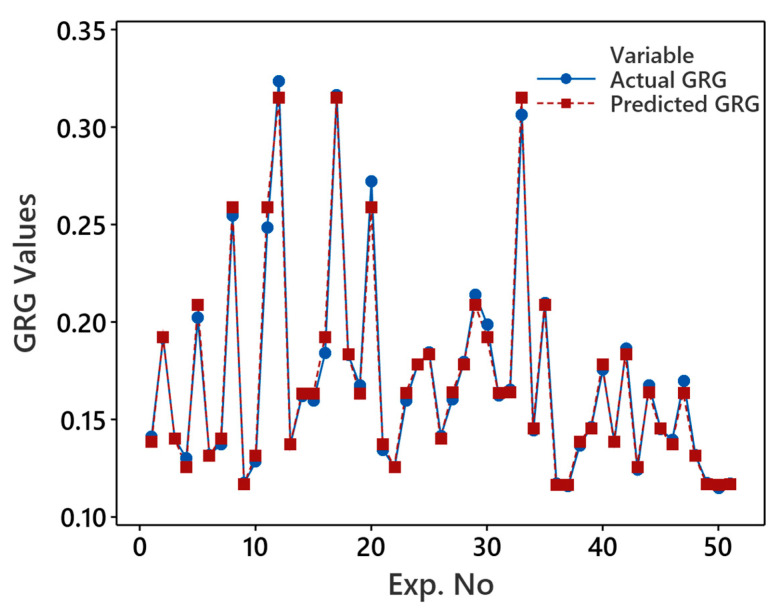
Comparison of actual and predicted GRG values.

The regression model, formulated based on GRG as per Equation (8), serves as the objective function in grey wolf optimization (GWO) to refine solution quality. Before its integration into GWO, it is important to measure its reliability and accuracy using statistical measures, particularly the coefficient of determination (R^2^), adjusted R^2^, predicted R^2^, and lack of fit. The R^2^ and adjusted R^2^ achieved are particularly high, at 98% and 97%, respectively, indicating a robust fit to the data and adequate explanatory power of retained model terms. This is further supported by the lack of fit, where the *p*-value exceeds the alpha value of 0.05, rendering it nonsignificant (0.739). The close agreement between actual and predicted GRG values, as illustrated in Figure 6, further validates the model’s accuracy and reliability in representing the underlying patterns in the data. Furthermore, the higher predicted R^2^ value (96%) highlights the model’s efficacy in accurately predicting printing parameter values within the defined level ranges.

Objective function based on GRG:

(18) $$\begin{array}{cc} Maximize,\;f\left(x\right) & =-0.1858+0.0089\;LT+0.000879\;NP+0.003206\;ID-0.000006\;FA\\ & -\;0.001007\;PS+0.000745\;ET+0.001014\;BT-0.000324\;BO-0.000002\;{FA}^{2}\\ & +\;0.000005\;{BO}^{2}-0.005038\;LT\times ID+0.003554\;LT\times FA-0.000012\;ID\times FA\\ & -\;0.000009\;ID\times BO \end{array}$$

Subject to constraints:

0.1≤LT≤0.3,2≤NP≤6,60≤ID≤100,0≤FA≤90,60≤PS≤80,260≤ET≤280,80≤BT≤100,0≤BO≤90

The maximum value of GRG obtained for optimal levels in Table 4 using Equation (8) is 0.320. To analyze whether the optimal levels obtained can be improved further, the metaheuristic approaches based on GWO are applied.

### 3.5. Grey Wolf Optimization

The steps followed for GWO are discussed in Section 2.5. Table 10 provides a detailed overview of the GWO algorithm’s performance across various parameter configurations. Particularly, the number of wolves in the population and the number of iterations significantly impact the optimization process, as evidenced by the objective function values obtained. For instance, when 10 wolves are used in the population, the objective function values range from 0.316 to 0.336 across different numbers of iterations. This suggests that while the algorithm with a smaller population size may converge faster (as seen with a value of 0.316 at 300 iterations), it may not always achieve the best solution, as demonstrated by the value of 0.336 at 250 iterations. Conversely, when employing a larger population size of 50 wolves, the objective function values range from 0.316 to 0.340. Here, we observe that increasing the population size does not necessarily guarantee better performance, as indicated by the slightly higher objective function values compared to those obtained with 10 wolves. Furthermore, analyzing the effect of the number of iterations reveals additional insights. For instance, consider the results obtained with 30 wolves: the objective function values fluctuate between 0.316 and 0.337 as the number of iterations increases from 100 to 300. This indicates that while increasing the number of iterations may lead to improvements in some cases (as seen with a decrease in the objective function value from 0.337 to 0.316 at 150 iterations), it may not always result in significant enhancements, as evidenced by the similar values obtained at 200 and 300 iterations (0.316 and 0.336, respectively).

**Figure 7:**
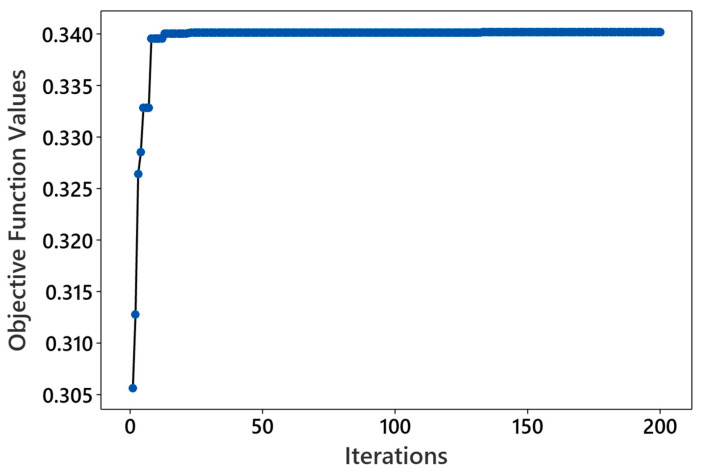
Convergence plots for 50 wolves and 200 iterations.

Figure 7 illustrates convergence plots obtained for the GWO, achieving its highest objective function value of 0.340 when 50 wolves are deployed over 200 iterations. The convergence plot demonstrates an upward trend, indicating effective improvement in solutions over successive iterations. The trajectory of the best objective function value reflects the dynamic nature of optimization, starting at 0.305 and showing substantial improvement to 0.339 by the 8th iteration. This initial phase of rapid enhancement suggests efficient navigation towards promising regions of the solution space. However, the objective remains almost consistent until the 12th iteration and then improves slightly to 0.340 by the 13th iteration, remaining constant thereafter until the maximum iteration of 200, indicating convergence.

Comparing the optimized printing parameters derived from GWO with those obtained using GRA reveals interesting findings. While most parameters align between the two optimization techniques, the discrepancy in the printing speed (60 mm/s) and bed temperature (100 °C) suggests that GWO may have discovered a more refined solution within the solution space that was not initially apparent through GRA. The optimal values of Ra, TS, and FS obtained are 4.63 µm, 88.5 MPa, and 103.12 MPa. This reflects good improvements in surface finish, tensile strength, and flexural strength compared to the initial optimal conditions, i.e., Ra = 5.55 µm, TS = 85.45 MPa, and FS = 98.36 MPa, respectively. These enhancements are indicative of the effectiveness of the optimization process in optimizing the printing parameters and enhancing the overall quality and mechanical performance of the printed components.

## 4. Practical Implications and Limitations of GWO

The above discussion reveals valuable insights into the practical implications of leveraging the GWO algorithm for refining printing parameters in additive manufacturing. The results suggest that the choice of population size and the number of iterations significantly influence the optimization process. Smaller population sizes may lead to faster convergence, but they may not always yield the best solution. Conversely, larger population sizes do not guarantee better performance. This indicates the importance of experimenting with different population sizes and iteration numbers to find the optimal balance between computational efficiency and solution quality.

Contrasting the results obtained from GWO with those obtained from other optimization techniques, such as grey relational analysis (GRA), highlights the strengths and weaknesses of each approach. While most parameters may align, discrepancies in certain parameters (such as printing speed and bed temperature) suggest that GWO may uncover more refined solutions within the solution space. This underscores the importance of exploring multiple optimization techniques to ensure robustness and reliability in the optimization process.

The optimized printing parameters derived from GWO result in improvements in surface finish, tensile strength, and flexural strength compared to initial optimal conditions. This indicates the effectiveness of the optimization process in enhancing the overall quality and mechanical performance of printed components. These enhancements can have significant practical implications, such as reducing material waste, improving product quality, and enhancing the durability of printed parts.

The findings suggest that GWO can be effectively utilized in industrial settings to optimize printing parameters for additive manufacturing processes. By fine-tuning printing parameters, manufacturers can achieve better performance, reduce production costs, and enhance the competitiveness of their products in the market. Additionally, the ability of GWO to discover refined solutions within the solution space highlights its potential for addressing complex optimization problems in various industrial domains beyond additive manufacturing.

Despite its effectiveness in solving optimization problems, GWO has several limitations and potential drawbacks to be considered. One significant limitation is its sensitivity to initial conditions, where small variations in the initial positions of wolves can lead to different optimization trajectories and outcomes. This sensitivity may result in suboptimal solutions or hinder the reproducibility of results across different runs or problem instances [58]. Additionally, like many metaheuristic algorithms, GWO is susceptible to the risk of premature convergence to local optima, especially in complex and multimodal optimization problems This can restrict the algorithm’s ability to explore the entire search space and find globally optimal solutions, particularly in problems with irregular objective functions [58,59,60]. Furthermore, the performance of GWO may heavily depend on the choice of algorithmic parameters, such as the number of iterations and the population size, which can make it challenging to achieve consistent results across different optimization tasks [61]. Thus, while GWO offers a powerful approach to optimization, researchers and practitioners should be aware of these limitations and carefully consider its applicability to specific problem domains.

## 5. Conclusions

The study systematically investigated the parametric optimization of the FDM process for PA12-CF parts by integrating response surface methodology (RSM), grey relational analysis (GRA), and grey wolf optimization (GWO). The research identified eight key process parameters and their relationships with response variables such as average surface roughness (Ra), tensile strength, and flexural strength. Through a definitive screening design (DSD) and subsequent analysis, significant impacts of parameters like layer thickness and build orientation on surface finish and mechanical properties were revealed. By consolidating responses into grey relational grades (GRG) and developing regression models, the study established a framework for optimizing printing parameters. GWO was then employed to refine the solution space and derive optimal parameter settings that significantly enhance the quality and mechanical properties of PA 12-CF parts, including a layer thickness (LT) of 0.1 mm, six perimeters (NP), infill density (ID) of 100%, fill angle (FA) of 0°, printing speed (PS) of 60 mm/s, extrusion temperature (ET) of 280 °C, bed temperature (BT) of 100 °C, and build orientation (BO) of 0°. These optimized parameters resulted in impressive improvements, with average surface roughness reduced to 4.63 µm, tensile strength enhanced to 88.5 MPa, and flexural strength increased to 103.12 MPa.

Furthermore, the comparison between GRA and GWO optimization techniques revealed the effectiveness of GWO in refining printing parameters and uncovering more refined solutions within the solution space. While both methods provided valuable insights, GWO demonstrated superior performance in enhancing surface finish and mechanical properties, particularly in optimizing parameters.

The practical implications of this research are significant for industrial applications of additive manufacturing. By implementing the optimized printing parameters derived from this study, manufacturers can achieve higher-quality PA 12-CF parts while reducing production costs and improving product competitiveness in the market. Additionally, the integration of advanced optimization techniques like GWO showcases the potential for addressing complex parametric optimization challenges across various industrial domains beyond additive manufacturing.

While the study provides valuable insights into parametric optimization, it is not without limitations. One limitation is the focus on a specific material (PA12-CF), which may limit the generalizability of the findings to other materials. Additionally, the research primarily explores the optimization of mechanical properties and surface finish, overlooking other important factors such as production time and energy consumption. Future research could address these limitations by expanding the scope to include a wider range of materials and considering additional optimization objectives to provide a more comprehensive understanding of FDM process optimization.

## Figures and Tables

**Table 1:** Levels of printing parameters of PA12-CF.

Printing Parameters	Symbol	Units	Levels		
			−1	0	1
Layer thickness	LT	mm	0.1	0.2	0.3
Number of perimeters	NP	-	2	4	6
Infill density	ID	%	60	80	100
Fill angle	FA	°	0	45	90
Printing Speed	PS	mm/s	60	70	80
Extrusion temperature	ET	°C	260	270	280
Bed temperature	BT	°C	80	90	100
Build orientation	BO	ͦ	0	45	90

**Table 2:** Experimental runs based on definitive screening design.

Exp. No	LT	NP	ID	FA	PS	ET	BT	BO	Exp. No	LT	NP	ID	FA	PS	ET	BT	BO
1	0.2	6	100	90	80	280	100	90	27	0.2	2	60	0	60	260	80	0
2	0.1	2	100	90	80	270	80	0	28	0.1	4	60	90	60	280	100	0
3	0.3	6	60	90	80	260	90	0	29	0.3	6	100	45	60	280	80	0
4	0.1	6	80	90	60	260	80	90	30	0.1	2	100	90	80	270	80	0
5	0.3	6	100	45	60	280	80	0	31	0.2	4	80	45	70	270	90	45
6	0.3	6	60	0	60	270	100	90	32	0.2	2	60	0	60	260	80	0
7	0.3	6	60	90	80	260	90	0	33	0.1	6	100	0	70	260	100	0
8	0.1	2	100	0	60	280	90	90	34	0.3	2	100	90	60	260	100	45
9	0.3	2	60	90	70	280	80	90	35	0.3	6	100	45	60	280	80	0
10	0.3	6	60	0	60	270	100	90	36	0.3	4	100	0	80	260	80	90
11	0.1	2	100	0	60	280	90	90	37	0.3	4	100	0	80	260	80	90
12	0.1	6	100	0	70	260	100	0	38	0.2	6	100	90	80	280	100	90
13	0.1	2	60	45	80	260	100	90	39	0.3	2	100	90	60	260	100	45
14	0.1	6	60	0	80	280	80	45	40	0.1	4	60	90	60	280	100	0
15	0.1	6	60	0	80	280	80	45	41	0.2	6	100	90	80	280	100	90
16	0.1	2	100	90	80	270	80	0	42	0.3	2	80	0	80	280	100	0
17	0.1	6	100	0	70	260	100	0	43	0.1	6	80	90	60	260	80	90
18	0.3	2	80	0	80	280	100	0	44	0.2	2	60	0	60	260	80	0
19	0.1	6	60	0	80	280	80	45	45	0.3	2	100	90	60	260	100	45
20	0.1	2	100	0	60	280	90	90	46	0.1	2	60	45	80	260	100	90
21	0.1	2	60	45	80	260	100	90	47	0.2	4	80	45	70	270	90	45
22	0.1	6	80	90	60	260	80	90	48	0.3	6	60	0	60	270	100	90
23	0.2	4	80	45	70	270	90	45	49	0.3	2	60	90	70	280	80	90
24	0.1	4	60	90	60	280	100	0	50	0.3	4	100	0	80	260	80	90
25	0.3	2	80	0	80	280	100	0	51	0.3	2	60	90	70	280	80	90
26	0.3	6	60	90	80	260	90	0									

**Table 3:** Measured average surface, tensile strength, and flexural strength based on definitive screening design.

Exp. No	Ra	TS	FS	Exp. No	Ra	TS	FS
1	15.00	45.02	53.22	27	11.26	49.28	57.39
2	8.07	56.25	64.39	28	8.06	50.93	59.74
3	15.33	43.79	54.28	29	12.93	65.98	77.38
4	14.56	38.24	43.96	30	7.70	56.51	67.53
5	12.20	64.29	72.69	31	13.39	52.86	61.99
6	19.95	46.26	52.12	32	10.99	50.73	59.49
7	15.90	43.10	53.15	33	3.85	71.16	83.46
8	9.14	71.12	80.57	34	16.78	48.73	57.15
9	17.06	32.33	37.92	35	11.96	64.55	75.70
10	20.60	45.10	50.22	36	20.36	36.62	42.95
11	8.82	69.56	80.00	37	20.55	35.81	42.00
12	4.37	73.72	86.46	38	15.49	44.98	49.04
13	14.55	42.52	49.72	39	15.80	48.58	56.98
14	10.93	49.28	57.79	40	8.45	50.24	58.92
15	11.22	50.30	55.34	41	14.67	43.29	50.77
16	8.69	55.05	62.17	42	17.16	62.44	73.23
17	4.51	73.16	85.79	43	16.00	36.16	42.40
18	17.16	61.78	72.32	44	10.66	51.20	60.05
19	10.54	50.97	59.78	45	15.62	47.89	56.16
20	9.80	72.48	84.96	46	14.32	43.55	50.51
21	14.42	41.13	46.60	47	12.50	54.50	63.91
22	15.91	37.19	42.75	48	19.95	46.42	51.68
23	12.94	51.14	60.32	49	16.38	31.50	36.63
24	7.63	49.32	57.84	50	20.72	35.29	41.38
25	17.17	62.01	72.72	51	16.79	31.93	36.64
26	15.22	45.14	54.29				

**Table 4:** Hyperparameter levels of GWO.

Hyperparameters	Level 1	Level 2	Level 3	Level 4	Level 5
Number of iterations	100	150	200	250	300
Number of wolves	10	20	30	40	50

**Table 5:** Analysis of variance for average surface roughness (Ra).

Source	F-Value	*p*-Value	Contribution
Linear	781.58	<0.001	86.79%
LT	2829.41	<0.001	52.36%
ID	150.11	<0.001	2.78%
FA	3.98	0.053	0.07%
PS	107.67	<0.001	1.99%
ET	72.89	<0.001	1.35%
BO	1525.44	<0.001	28.23%
Square	137.65	<0.001	10.87%
ID^2^	189.39	<0.001	3.70%
PS^2^	234.1	<0.001	7.17%
Two-way Interactions	43.39	<0.001	1.61%
LT × BO	40.88	<0.001	1.15%
ET × BO	24.62	<0.001	0.46%
Error			0.74%
Lack-of-Fit	0.72	0.634	0.08%
Pure Error			0.66%
Total			100.00%

**Table 6:** Analysis of variance for tensile strength (TS).

Source	F-Value	*p*-Value	Contribution
Linear	886.5	<0.001	87.43%
LT	494.9	<0.001	6.97%
ID	1409.17	<0.001	19.85%
FA	1332.43	<0.001	18.77%
PS	252.2	<0.001	3.55%
ET	439.95	<0.001	6.20%
BT	359.87	<0.001	5.07%
BO	1917.01	<0.001	27.01%
Square	139.26	<0.001	4.72%
ET^2^	125.7	<0.001	0.15%
BT^2^	220.75	<0.001	4.57%
Two-way Interactions	173.05	<0.001	7.31%
ID × BO	302.79	<0.001	5.45%
FA × PS	117.63	<0.001	1.10%
FA × ET	54.29	<0.001	0.76%
Error			0.54%
Lack-of-Fit	0.56	0.696	0.03%
Pure Error			0.50%
Total			100.00%

**Table 7:** Analysis of variance for flexural strength (FS).

Source	F-Value	*p*-Value	Contribution
Linear	433.52	<0.001	86.21%
LT	217.19	<0.001	6.17%
ID	679.03	<0.001	19.29%
FA	596.98	<0.001	16.96%
PS	102.08	<0.001	2.90%
ET	172.14	<0.001	4.89%
BT	177.29	<0.001	5.04%
BO	1089.92	<0.001	30.96%
Square	94.12	<0.001	6.07%
ET^2^	81.38	<0.001	0.03%
BT^2^	152.09	<0.001	6.05%
Two-way Interactions	77.85	<0.001	6.64%
ID×BO	148.46	<0.001	5.25%
FA×PS	44.12	<0.001	0.84%
FA× ET	19.18	<0.001	0.54%
Error			1.08%
Lack-of-Fit	0.47	0.761	0.06%
Pure Error			1.02%
Total			100.00%

**Table 8:** Computation of grey relational coefficients and grey relational grade.

Exp. No	GRC_Ra_	GRC_TS_	GRC_FS_	GRG	Exp. No	GRC_Ra_	GRC_TS_	GRC_FS_	GRG
1	0.142	0.140	0.141	0.141	27	0.176	0.153	0.152	0.160
2	0.220	0.181	0.175	0.192	28	0.220	0.159	0.159	0.179
3	0.140	0.136	0.144	0.140	29	0.159	0.242	0.242	0.214
4	0.145	0.123	0.122	0.130	30	0.227	0.182	0.188	0.199
5	0.166	0.228	0.213	0.202	31	0.155	0.166	0.166	0.162
6	0.113	0.143	0.139	0.132	32	0.179	0.158	0.158	0.165
7	0.136	0.135	0.141	0.137	33	0.330	0.294	0.295	0.306
8	0.203	0.294	0.267	0.255	34	0.130	0.151	0.152	0.144
9	0.129	0.111	0.112	0.117	35	0.168	0.230	0.230	0.210
10	0.111	0.140	0.134	0.128	36	0.112	0.120	0.120	0.117
11	0.208	0.276	0.262	0.248	37	0.111	0.118	0.119	0.116
12	0.311	0.330	0.330	0.324	38	0.139	0.140	0.132	0.137
13	0.145	0.133	0.133	0.137	39	0.137	0.151	0.151	0.146
14	0.179	0.153	0.153	0.162	40	0.214	0.156	0.157	0.176
15	0.176	0.156	0.147	0.160	41	0.145	0.135	0.136	0.138
16	0.210	0.175	0.167	0.184	42	0.128	0.215	0.216	0.186
17	0.306	0.321	0.321	0.316	43	0.135	0.119	0.119	0.124
18	0.128	0.211	0.213	0.183	44	0.183	0.160	0.160	0.167
19	0.184	0.159	0.159	0.167	45	0.138	0.148	0.149	0.145
20	0.194	0.312	0.311	0.272	46	0.147	0.136	0.135	0.139
21	0.146	0.130	0.127	0.134	47	0.163	0.173	0.173	0.170
22	0.136	0.121	0.120	0.125	48	0.113	0.144	0.138	0.132
23	0.159	0.159	0.161	0.160	49	0.133	0.110	0.110	0.118
24	0.228	0.153	0.154	0.178	50	0.110	0.117	0.118	0.115
25	0.128	0.212	0.213	0.184	51	0.130	0.111	0.110	0.117
26	0.141	0.140	0.144	0.142					

**Table 9:** Optimal levels for process parameters.

Process Parameters	Levels			Optimal Levels
	−1	0	1	
Layer thickness	0.196	0.156	0.149	−1 (0.1 mm)
Number of perimeters	0.171	0.153	0.175	1 (6)
Infill density	0.147	0.158	0.196	1 (100%)
Fill angle	0.190	0.170	0.148	−1 (0°)
Print speed	0.173	0.199	0.153	0 (70 mm/s)
Extrusion temperature	0.163	0.162	0.178	1 (280 °C)
Bed temperature	0.155	0.187	0.176	0 (90 °C)
Build orientation	0.197	0.157	0.146	−1 (0°)

**Table 10:** Combination of GWO parameters and objective function values.

S. No	No of Wolves	No of Iterations	Objective Function Values
1	10	100	0.323
2	10	150	0.336
3	10	200	0321
4	10	250	0.336
5	10	300	0.316
6	20	100	0.316
7	20	150	0.316
8	20	200	0.316
9	20	250	0.319
10	20	300	0.316
11	30	100	0.316
12	30	150	0.320
13	30	200	0.316
14	30	250	0.337
15	30	300	0.336
16	40	100	0.316
17	40	150	0.337
18	40	200	0.337
19	40	250	0.337
20	40	300	0.337
21	50	100	0.320
22	50	150	0.337
23	50	200	0.340
24	50	250	0.337
25	50	300	0.337

## Data Availability

Data are contained within the article.
